# Bruton’s tyrosine kinase inhibition in ITP: Wayrilz (rilzabrutinib) as a disease-modifying strategy

**DOI:** 10.1097/MS9.0000000000004164

**Published:** 2025-10-28

**Authors:** Syeda Fadak Zahra Hujjat, Harshika Khaim Chandani, Muhammad Saad Khan, Devya Khaim Chandani, Abubakr Mahmoud

**Affiliations:** a Department of Medicine, Allama Iqbal Medical College, Lahore, Pakistan; b Department of Medicine, Jinnah Sindh Medical University, Karachi, Pakistan; c Department of Medicine, Shaheed Mohtarma Benazir Bhutto Medical College (SMBBMC), Karachi, Pakistan; d Department of Medicine, University of Khartoum, Khartoum State, Sudan

**Keywords:** autoimmune hematology, Bruton’s tyrosine kinase, disease-modifying therapy, immune thrombocytopenia, rilzabrutinib

## Abstract

Immune thrombocytopenia (ITP) is an acquired autoimmune disorder characterized by immune-mediated platelet destruction and impaired platelet production, leading to bleeding risk, morbidity, and reduced quality of life. Incomplete responses, adverse effects, and relapses are the limitations of current treatments, which include corticosteroids, intravenous immunoglobulin, rituximab, and thrombopoietin receptor agonists. On 29 August 2025, the U.S. Food and Drug Administration (FDA) approved Wayrilz (rilzabrutinib), a first-in-class oral Bruton’s tyrosine kinase inhibitor, for the treatment of adult patients with chronic or persistent ITP who have had an insufficient response to previous treatments. Through modulation of both Fc receptor and B-cell receptor signaling, the agent aims to address disease pathophysiology as a treatment that is disease-modifying in nature rather than simplistically dealing with symptoms. Clinical trials, including the key LUNA 3 trial, have shown good efficacy and safety, with durable platelet responses and minimal severe adverse events. This editorial examines rilzabrutinib’s scientific justification, clinical data, comparative analysis, side effects, financial ramifications, and potential applications in the treatment of ITP.

## Background

Immune thrombocytopenia (ITP) is an acquired autoimmune disease marked by low platelet counts due to accelerated immune-mediated destruction and impaired megakaryocyte function^[1]^. Mucocutaneous bleeding, petechiae, and bruising are common. In severe cases, hemorrhage can be seen that is life-threatening^[2]^. Chronic course of the disease is noted in about two-thirds of the cases. The estimated annual incidence is 3–4 per 100 000 population^[3]^. Chronic ITP means repeat episodes of bleeding and result in dependency on immunosuppressive therapies besides psychological stress and added burden on healthcare. This manuscript has been developed in accordance with the TITAN Guidelines (2025)^[4]^.

Conventional therapies such as corticosteroids, rituximab, intravenous immunoglobulin, and thrombopoietin receptor agonists (TPO-RAs) improve platelet counts but are hindered by relapses, adverse events, and incomplete responses^[5]^. Therefore, novel approaches targeting the underlying immunopathology are required.

**HIGHLIGHTS**

- Paradigm shift in ITP therapy: Discusses the recent FDA approval of rilzabrutinib, the first oral BTK inhibitor for ITP, representing a move from broad immunosuppression to targeted, disease-modifying treatment.
- Novel mechanism of action: Explains how dual inhibition of FcγR and B-cell receptor signaling uniquely addresses both platelet destruction and autoantibody production, targeting the core pathophysiology of ITP.
- Durable efficacy and favorable safety: Highlights robust clinical trial data (LUNA 3) demonstrating sustained platelet responses with a low incidence of severe adverse events, offering a promising benefit–risk profile.
- Positioning in the treatment landscape: Compares rilzabrutinib favorably to existing therapies (TPO-RAs, rituximab), emphasizing its oral convenience, potential for disease modification, and improved tolerability.
- Broad future implications: Considers the drug’s potential to reduce healthcare costs, its future applications in pediatric and treatment-naïve patients, and its role as a pioneer for BTK inhibition in other autoimmune diseases.

On 29 August 2025, the FDA approved Wayrilz (rilzabrutinib) for adult patients with persistent or chronic ITP who had an inadequate response to prior therapies^[6]^. This is a paradigm shift, because up till now patients were offered only oral medications that could serve as mere add-on agents to increase platelet counts but not directly address the core problem of immune dysregulation.

## Mechanism of action and pharmacokinetics

Rilzabrutinib is a reversible, selective inhibitor of Bruton’s tyrosine kinase (BTK) that acts on both B lymphocytes and myeloid cells^[7]^. By modulating B-cell receptor and Fc receptor signaling, it decreases the formation of autoantibodies as well as Fc-mediated platelet phagocytosis by macrophages^[8]^. Rilzabrutinib minimizes systemic immune compromise by targeting particular disease-driving pathways, in contrast to traditional immunosuppressants that generally reduce immune activity.

According to pharmacokinetic research, oral rilzabrutinib is rapidly absorbed, reaching steady-state concentrations in a matter of days^[9]^. It undergoes hepatic metabolism, accumulates little, and has not been linked to any significant drug–drug interactions in clinical trials. Twice-daily dosing is supported by the comparatively short half-life, which strikes a balance between tolerability and effectiveness.

## Dosing and administration

The recommended dose of Wayrilz is 400 mg orally twice daily taken on an empty stomach^[6]^. This oral schedule confers a practical advantage over intravenous therapies such as rituximab and frequent injections associated with TPO-RAs. Side effects seen in trials were predominantly low-grade (e.g., gastrointestinal upset, fatigue and mild infections)^[8]^. Crucially, rates of serious infections and bleeding were low, as were overall cessation rates.

## Evaluation of safety and efficacy through clinical trials

Preliminary data came from a Phase 1/2 trial with 60 highly pretreated ITP patients – the results of which will be submitted for publication soon. About 40% had platelets ≥30 × 10⁹/L at various time points, with durable responses observed at 24 weeks^[8]^. The LUNA 3 Phase 3 study compared rilzabrutinib to placebo in patients with chronic ITP that had an insufficient response to prior treatment. It had significantly higher stable platelet responses at 6–8 weeks and fewer bleedings^[9]^. This was supported by data in a Phase 2 open-label extension study, where long-term safety and efficacy were reported for >1 year, with no safety signals^[10]^. Taken together, these trials demonstrate rilzabrutinib as an active and well-tolerated disease-modifying treatment in refractory ITP.

## Comparative perspective

Current second-line ITP therapies include TPO-RAs (eltrombopag and romiplostim) and rituximab. While TPO-RAs enhance platelet production, they do not address the immune-driven platelet destruction and require long-term use^[4]^. Rituximab targets B cells but is associated with prolonged immunosuppression, risk of infections, and limited durability of response^[11]^. While in contrast, rilzabrutinib provides oral administration, disease modification, and a favorable safety profile. Its positioning in the treatment algorithm is likely between TPO-RAs and rituximab, especially for the patients who do prefer oral therapy or the ones who fail conventional regimens.

## Complications

Adverse events reported with rilzabrutinib were primarily mild to moderate. The most common were gastrointestinal symptoms, fatigue, and upper respiratory infections^[8,9]^. Importantly, unlike other immunosuppressants, rilzabrutinib did not significantly increase severe infections or hemorrhagic risk^[10]^. One needs to keep watching out for problems even after rilzabrutinib hits market. Long-term use of the BTK inhibitors could mess with how a person’s body fights some infections (like making their immune system weaker) but so far those issues have not popped up^[12]^.

## Cost effectiveness and future implications

Cost stays an important consideration in ITP management. Treating ITP is not cheap already. The TPO-RAs cost a lot, but rilzabrutinib, which you can take by a mouth, might wind up being more cost-friendly. It could cut how often people need to go to the hospital, get blood transfusions or stay on heavy duty immunosuppressive therapy^[13]^. Looking ahead, folks are interested for testing rilzabrutinib for kids with ITP (not many good choices there). Maybe it will work for people earlier in their disease too, could maybe even change how things play out. The BTK signaling messes things up in other autoimmune conditions like hemolytic anemia and pemphigus vulgaris conditions^[14]^. Real-world registry data will be critical to assess its comparative cost-effectiveness, long-term tolerability, and patient-reported outcomes.

## Limitations and contraindications

Although rilzabrutinib offers a major therapeutic promise, certain constraints must be acknowledged. Investigations to date have predominantly involved adults with refractory ITP, leaving data in pediatric or treatment-naïve cohorts yet to emerge^[10]^. Known contraindications involve hypersensitivity reactions related to compound or its formulation components, while heightened vigilance is recommended for individuals exhibiting severe hepatic dysfunction^[6]^.

In addition, concurrent use with other targeted pharmacological agents remains under an evaluation for safety. Infrequent but possible concerns such as immunologic instability necessitate ongoing monitoring following market introduction.

## Conclusion

The regulatory approval of Wayrilz (rilzabrutinib) signifies an advancement within the ITP therapeutics, moving management away from broad-spectrum immunosuppression toward more selective immune modulation strategies. Its administration by mouth, modification of disease pathophysiology, and apparently positive safety characteristics position it as a beneficial option – especially among patients with the restricted access to alternative interventions. As applies to any new pharmaceutical agent, extended outcome data along with observational evidence and economic impact assessments will ultimately inform its clinical niche. Should efficacy together with tolerability persist over time, rilzabrutinib may transform established practice in autoimmune hematology and potentially help further use of the BTK inhibition models throughout varied autoimmune conditions.

## Data Availability

No data were generated for this manuscript.

## References

[1] Cines DB Blanchette VS. Immune thrombocytopenic purpura. N Engl J Med 2002;346:995–1008. 11919310 10.1056/NEJMra010501

[2] Neunert C Terrell DR Arnold DM. American society of hematology 2019 guidelines for ITP. Blood Adv 2019;3:3829–66. 31794604 10.1182/bloodadvances.2019000966 PMC6963252

[3] Rodeghiero F Stasi R Gernsheimer T. Standardization of terminology, definitions and outcome criteria in ITP. Blood 2009;113:2386–93. 19005182 10.1182/blood-2008-07-162503

[4] Agha RA Mathew G Rashid R, TITAN Group. Transparency in the Reporting of Artificial Intelligence – the TITAN guideline. Prem J Sci 2025;10:100082. doi:10.70389/PJS.100082.

[5] Provan D Arnold DM Bussel JB. Updated international consensus report on ITP management. Blood Adv 2019;3:3780–817. 31770441 10.1182/bloodadvances.2019000812 PMC6880896

[6] U.S. Food and Drug Administration. FDA approves Wayrilz (rilzabrutinib) for chronic ITP. FDA News Release. 2025.

[7] Smith P. Bruton tyrosine kinase inhibition in autoimmune disease. Nat Rev Rheumatol 2022;18:317–29.

[8] Kuter DJ Efraim M Mayer J. Rilzabrutinib, an oral BTK inhibitor, in ITP. N Engl J Med 2022;386:1421–31. 35417637 10.1056/NEJMoa2110297

[9] Bussel JB. LUNA 3: phase 3 trial of rilzabrutinib in chronic ITP. Blood 2024;144:122–24.38963668

[10] Mahévas M. Long-term efficacy and safety of rilzabrutinib in ITP. Haematologica 2025;110:745–53.

[11] Ghanima W. Rituximab as second-line treatment for adult ITP. Blood 2012;119:5640–48.22535666

[12] Byrd JC. Long-term safety of BTK inhibitors in B-cell malignancies. Blood 2019;133:1578–89.

[13] Chugh SS. Economic burden of chronic ITP. J Med Econ 2020;23:1580–88.

[14] Khodadadi L Cheng Q Radbruch A. BTK inhibitors in autoimmunity. Trends Pharmacol Sci 2021;42:573–86.

